# Current Advances in Classification, Prediction and Management of Microvascular Invasion in Hepatocellular Carcinoma

**DOI:** 10.1111/jcmm.70746

**Published:** 2025-07-30

**Authors:** Zhenli Li, Lindi Xu, Shuaishuai Zhu, Xingshun Qi, Wei Zhang, Yufu Tang

**Affiliations:** ^1^ Department of Hepatobiliary Surgery General Hospital of Northern Theater Command Shenyang China; ^2^ Department of General Surgery The 963rd Hospital of the Joint Service Support Force of the PLA Jiamusi China; ^3^ Dalian Medical University Dalian China; ^4^ Department of Gastroenterology General Hospital of Northern Theater Command Shenyang China

**Keywords:** classification, hepatocellular carcinoma, microvascular invasion, prediction, recurrence

## Abstract

Liver resection remains the mainstay curative treatment for hepatocellular carcinoma (HCC); however, the recurrence rate is reported to exceed 70% within 5 years after surgery. Microvascular invasion (MVI) has attracted great research interest in the last decade and has been confirmed to be an independent risk factor for postoperative recurrence and survival. Presently, the diagnosis of MVI depends on pathological specimens, which are not helpful in guiding preoperative planning and intraoperative decision‐making. However, preoperative MVI prediction has developed rapidly with the widespread application of predictive models. Besides the well‐defined clinical predictive factors, radiomics and artificial intelligence (AI)‐based models can provide accurate predictions of MVI. In terms of the specific management of MVI, multiple pre‐ and intraoperative therapeutic choices have shown favourable effects in patients at high risk of MVI indicated by predictive models. Several postoperative adjuvant therapies were also demonstrated to be associated with improved surgical outcomes in patients diagnosed with MVI. Considering that the present perspectives concerning MVI‐related management are still controversial, based on the latest research, the present paper provides updated insights into the **1**) diagnosis and classification of MVI, **2**) the predictive factors and models of MVI and **3**) effective therapeutic choices of MVI in pre‐, intra‐ and postoperative processes. The remaining challenges in the management of MVI are highlighted to stimulate further explorations of the precise and individualised management of MVI.

## 1 Introduction

Hepatocellular carcinoma (HCC) ranks as the fifth most common malignancy and the fourth leading cause of cancer‐related death worldwide, with more than 600,000 deaths reported globally each year [1, 2]. Liver resection is the cornerstone of curative‐intent HCC treatment; however, the recurrence rate is reported to exceed 70% within 5 years after surgery, even in patients identified as optimal candidates for curative treatment [3]. Since primary HCC tends to invade surrounding vessels, haematogenous metastasis is considered a potential mechanism contributing to postoperative recurrence [4]. In the past, HCC with major vascular invasion has been recognised as a contraindication for liver resection and is associated with significantly increased risks of postoperative recurrence and mortality [5]. Lately, microvascular invasion (MVI) has attracted research interest among surgeons, and clinical evidence has confirmed it as an independent risk factor for postoperative recurrence and survival [6]. As indicated by the multicentre database, Sheng et al. [7] showed that the 3‐year recurrence rates were 62.5%, 71.6%, and 86.1% in M0, M1, and M2 groups, respectively, with an elevated recurrence risk associated with the severity of MVI. Thus, an early assessment of MVI status, as well as a standard classification system that is convenient for formulating rational treatment, is becoming increasingly crucial for hepatobiliary surgeons. First, an accurate prediction of MVI could guide preoperative treatment and intraoperative decision‐making to optimise surgical outcomes. Second, an ideal MVI classification system could stratify patients with varied recurrence risks, thus providing effective therapies to improve the long‐term prognosis [8].

The present MVI diagnosis depends on pathological specimens, and the MVI classification varies between countries, which is not helpful in guiding perioperative procedures [9]. The preoperative assessment and prediction of MVI status have attracted attention with the rapid development of diagnostic tools and the widespread application of predictive models [10, 11]. Several clinical factors have been proved useful in predicting MVI, such as patient and tumour characteristics, imaging features and laboratory biomarkers [12]. Furthermore, the emergence of radiomics and models based on artificial intelligence (AI) could provide accurate MVI predictions [13]. Among these models, a predictive nomogram incorporating seven independent clinical variables was proposed by Lei et al. [14] and exhibited excellent performance in predicting MVI, with a positive/negative predictive value of 57.9%/83.2%. It was used to identify patients at high risk of MVI in a randomised clinical trial to explore the impact of neoadjuvant radiotherapy on surgical outcomes of patients with HCC [15]. However, most of these predictive models are rarely utilised in clinical practice due to the undesirable performance in external validation cohorts. Several issues concerning the interpretability, repeatability and generalisability of these models also remain to be addressed in future research. In terms of the specific management of MVI, several pre‐ and intraoperative therapeutic choices have shown favourable effects in patients at high risk of MVI as indicated by predictive models [15, 16], and postoperative adjuvant therapies have also been demonstrated to be associated with improved surgical outcomes in patients diagnosed with MVI [17, 18]. However, the optimal therapeutic protocols of MVI are still under debate and warrant in‐depth explorations.

Given the advances and controversies focusing on the classification, prediction and management of MVI, the present paper provides updated insights into the **1**) diagnosis and classification of MVI, **2**) the predictive factors and models of MVI and **3**) effective therapeutic choices of MVI in pre‐, intra‐ and postoperative processes. The challenges in the management of MVI are highlighted to stimulate further explorations on the precise and individualised management of HCC and MVI.

## 2 Diagnosis and Classification of MVI

The present diagnosis and classification of MVI are based on pathological specimens. In the past few years, pathological teams worldwide have proposed several practical MVI grading systems to guide prognosis prediction and postoperative treatment (Table 1). Fujita et al. [20] and Iguchi et al. [23] from Japan considered the presence of multiple invaded portal venous vessels ( ≥ 2) and more than 50 invading carcinoma cells as essential indicators of MVI. Another Japanese research team [19] established an MVI grading system using the number of MVI and successfully stratified patients with varied recurrence risks after hepatectomy. Roayaie et al. [21], from the United States, defined the invasion of a vessel with a muscular wall and invasion of a vessel > 1 cm from the tumour as risk features of MVI. They incorporated the risk features into a scoring system to accurately determine recurrence risk after hepatectomy in patients with HCC and MVI. The MVI grading criteria proposed by the Chinese team are classified according to the number and distribution of MVI [7]. The criteria (version 2024) [22] classified MVI as follows: M0: no MVI; M1: 1–5 MVIs, all in the tumour‐adjacent tissues (proximal MVI, ≤ 1 cm away from the tumour boundary); M2a: > 5 MVIs, all in tumour‐adjacent tissues; and M2b: ≥ 1 MVI, > 1 cm away from the tumour boundary. Upon in‐depth comparative analysis, the advantages and disadvantages of these four classification systems could be concluded as follows (Table 1): (1). In the core definition of MVI, the location and vessel type of MVI were defined as tumour‐adjacent portal tract by these systems, and the Chinese system specifically proposed > 1 cm away from the tumour boundary as an indicator of severe MVI. As for the morphology, only the systems of Fujita et al. and Wang et al. confirmed the definition of cancer cell clusters. For the number of MVI, Sumie et al. Fujita et al. and Wang et al. defined the number of invaded vessels and invading HCC cells as indicators of severe MVI, while Roayaie et al. did not mention it. (2). Regarding study populations, only the system of China constituted a multicentre, large‐sample cohort, while other studies were single‐centre investigations. (3). In prognostic stratification power, all four classification criteria effectively differentiated surgical outcomes, which could serve as potential prognostic indicators. Especially, the system of China demonstrated significantly superior discriminative power in long‐term prognosis compared to the traditional 2‐tiered MVI classification. 4). For clinical utility, Wang's classification system based on the seven‐point baseline sampling protocol offered substantial feasibility in both qualitative and quantitative assessments, which have become the most widely adopted approach in Chinese medical centres. In summary, the classification criteria of China demonstrated clear advantages in core definition standards, study cohort, prognostic stratification, and clinical utility, which merit widespread implementation. Unfortunately, none of the above criteria have undergone robust external validation to confirm the validity. Thus, the globally standardised classification criteria still demand further high‐quality studies.

**TABLE 1:** Comparisons of current MVI grading systems worldwide.

Research	The MVI definition	The MVI grading	Prognostic stratification power	Extent of validation
Sumie et al. [19] Japan	Location: portal and hepatic vein of the surrounding liver tissue contiguous with the tumour edge. Number: NA Cancer cell cluster: NA Vessel type: portal and hepatic vein	Mild MVI group: 1–5 invaded vessels Severe MVI group: > 5 invaded vessels	Great stratification power in RFS and DFS	Cohort: a single‐centre database of 207 patients Validation: none
Fujita et al. [20] Japan	Location: portal vein accompanied with hepatic artery and bile duct in the portal tract Number: 50 invading carcinoma cells Cancer cell cluster: yes Vessel type: portal tract	High‐MVI group: multiple invaded portal venous vessels (≥ 2) and ≥ 50 invading carcinoma cells Low‐MVI group: single invaded portal venous vessel or < 50 invading carcinoma cells	Great stratification power in OS and DFS	Cohort: a single‐centre database of 280 patients Validation: none
Roayaie et al. [21] America	Location: tumour within a vascular space lined by endothelium Number: NA Cancer cell cluster: NA Vessel type: NA (risk factors: invasion of a vessel with a muscular wall and invasion of a vessel ≥ 1 cm)	A: none B1: MVI with no risk factors B2: MVI with 1 risk factor B3: MVI with 2 risk factors	Great stratification power in OS and RFS	Cohort: a single‐centre database of 131 patients Validation: none
Wang et al. [22] China	Location: tumour‐adjacent liver tissue Number: 5 sites of MVI Cancer cell cluster: yes Vessel type: portal, hepatic and tumour capsular vessels	M0: no MVI M1: 1–5 sites of MVI in the tumour‐adjacent liver tissue ≤ 1.0 cm away from the main tumour, M2a: > 5 sites of MVI in the tumour‐adjacent liver tissue ≤ 1.0 cm M2b: any MVI in distant liver tissue > 1.0 cm away from the tumour	Great stratification power in OS, RFS and early RFS	Cohort: a multicentre database of 1546 patients Validation: none

Abbreviations: DFS, disease‐free survival; MVI, microvascular invasion; NA, not applicable; OS, overall survival; RFS, recurrence‐free survival.

In addition, it is hard to meet the comprehensive therapeutic requirements of HCC patients using only pathological classification in clinical practice. The clinical classification combining pathological and clinical features showed superior performance in predicting postoperative prognosis and better specified the appropriate adjuvant therapy. Recently, Wang et al. [24] established a practical scoring system that combined the alpha‐fetoprotein (AFP) level, tumour number, tumour diameter and MVI features, and categorised patients with MVI into class A (≤ 3 points), class B (3.5 – 5 points) and class C (> 5 points) according to a risk assessment. The prognostic outcomes demonstrated that this classification shows favourable performance in predicting postoperative recurrence and that only patients with class C could benefit from adjuvant transcatheter arterial chemoembolisation (TACE). In addition, an MVI classification system [25] proposed by Ma et al. incorporated significant imaging variables like arterial phase peritumoral enhancement, boundary of the tumour enhancement, tumour necrosis stratification and boundary of the necrotic area. The imaging‐based classification could differentiate MVI‐positive patients and facilitate the precise selection of candidates who could benefit from adjuvant hepatic arterial infusion chemotherapy (HAIC) treatment. Given that most MVI‐related classifications are based on retrospective data, further high‐quality, prospective clinical research is warranted for more precise clinical classifications.

## 3 Predictive Factors and Models

**FIGURE 1:**
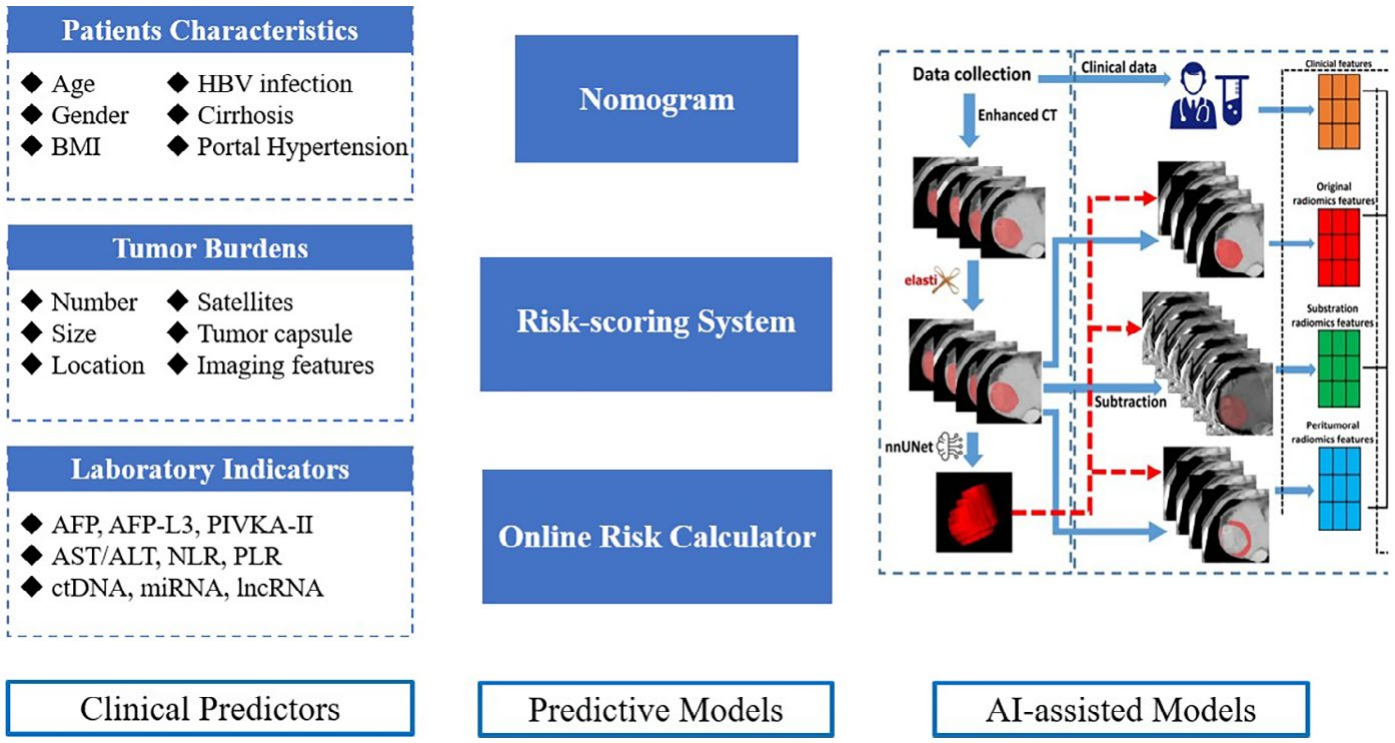
The development of MVI prediction strategies, including clinical predictors, predictive models and AI‐assisted models. MVI, microvascular invasion; AI, artificial intelligence. Reproduced with permission [26], Copyright 2024, Springer.

MVI is one of the most vital pathological indicators used for postoperative surveillance and therapy to decrease the recurrence risk of HCC. Nevertheless, due to the lag caused by a pathological diagnosis, it cannot be used in pre‐ and intraoperative processes. Recently, the accurate prediction of MVI before surgery has inspired many clinical studies. The present predictors of MVI can be classified as follows: (1) clinical variables, (2) imaging features and (3) predictive models (Figure 1).

### 3.1 Clinical Variables

Clinical variables are the most frequently utilised indicators among surgeons to predict the presence of MVI and estimate recurrence risk after hepatectomy for HCC. They can be categorised into clinical characteristics, tumour features, laboratory parameters and molecular biomarkers.

#### 3.1.1 Clinical Characteristics

Some clinical characteristics, such as age, gender, body mass index, hepatitis B virus (HBV) infection, cirrhosis and portal hypertension [27, 28], were identified to be independent risk factors of MVI and have been incorporated into models to enhance their predictive performance [14, 24].

#### 3.1.2 Tumour Features

Researchers found that aggressive tumour burdens, such as larger tumour diameter, multiple tumours and the presence of invaded tumour capsules, were associated with a significantly higher incidence of MVI. Other tumour features like tumour location and satellites were also strongly correlated with MVI [29].

#### 3.1.3 Laboratory Parameters

Laboratory parameters, such as inflammation‐related indicators (e.g., neutrophil‐to‐lymphocyte ratio, platelet‐to‐lymphocyte ratio, aspartate aminotransferase/alanine aminotransferase ratio and γ‐glutamyl transferase levels) and tumour‐related biomarkers (e.g., AFP, AFP‐L3 and protein induced by vitamin K absence or antagonist II) were demonstrated to be indicators of MVI with excellent predictive performance [30].

#### 3.1.4 Molecular Biomarkers

In recent years, with improvements in next‐generation sequencing and liquid biopsy technology, a growing number of molecular biomarkers have been applied to predict MVI with ideal performance.

##### 3.1.4.1 DNA‐Related Biomarkers

With a deeper understanding of MVI at the genetic level, several DNA‐related biomarkers associated with increased risks of MVI were reported. Beaufrère et al. [31] identified a six‐gene signal (*FAS*, *ANGPTL7*, *UGT2B7*, *ROS1, MKI67* and *GMNN*) that was closely correlated with MVI. Analysis showed that the six‐gene signal had sensitivity, specificity and area under the curve (AUC) values of 82%, 81% and 0.82, respectively, in predicting the presence of MVI. The signal also indicated decreased overall survival (OS) in patients with HCC diameters of ≤ 3 cm. Cai et al. [32] found that the expression of stathmin 1 (*STMN1*) might be a potent biomarker of MVI, and the level of *STMN1* was positively correlated with the incidence of MVI.

Circulating tumour DNA (ctDNA) is an emerging technology with unique noninvasive and highly sensitive and specific properties, which has gained great potential to detect and predict MVI status preoperatively [33]. By the targeted sequencing of 1021 solid tumour genes in preoperative peripheral blood and intraoperative tumour tissues, Wang et al. [33] identified that a ctDNA variant allele frequency (VAF) of > 0.83% was independently associated with the presence of MVI, with sensitivity and specificity of 78.6% and 81.8% respectively. Subsequent research showed that a maximal VAF of > 0.018 was also associated with MVI, with an AUC of 0.85 [34].

##### 3.1.4.2 RNA‐Related Biomarkers

MicroRNAs (miRNAs) are noncoding RNAs with important regulatory functions in the progression of HCC. Their excellent stability and detectability in plasma make them a perfect noninvasive biomarker for predicting MVI [35]. Researchers recently demonstrated that miRNAs, including miR‐125b and miR‐497, could serve as potential predictors of MVI, with favourable predictive performance [36, 37].

Long noncoding RNA (lncRNA) is another type of noncoding RNA and participates in HCC progression through various mechanisms [38]. Ma et al. [39] found that a new type of lncRNA, AC104958.2, was downregulated 1.8‐fold in MVI‐negative HCC tissues compared to MVI‐positive tissues by assessing HCC samples and adjacent nontumoral tissues. Another research team showed that the high expression of lnc‐TSPAN12 differed between MVI‐positive and normal liver tissue, and it could significantly discriminate MVI with an AUC of 0.855, suggesting that lnc‐TSPAN12 could serve as a diagnostic biomarker of MVI [35].

### 3.2 Imaging Features and Radiomics

The prediction of MVI based on imaging features has become a research hotspot in recent years, especially with the rapid development of radiomics [40]. The conventional imaging features in predicting MVI include: 1) computed tomography (CT) imaging of invaded tumour capsules, nonsmoothness and enhancement of the tumour edge, CT value of the arterial phase and intratumoral blood supply during the multiple phases [13], 2) magnetic resonance imaging (MRI) of the specific apparent diffusion coefficient, elastic imaging characteristics and limited spectrum imaging characteristics [41] and 3) other imaging modalities, such as the standardised uptake value (SUV) of positron emission tomography–CT (PET‐CT) and ultrasound elastography characteristics [42].

#### 3.2.1 CT Radiomics

Conventional CT features provide relatively few metrics for MVI prediction; however, CT radiomics can transform raw images into quantitative features and reveal tumour pathophysiology in a high‐throughput manner [43]. Therefore, CT radiomics are expected to provide more possibilities for predicting MVI. A recent systematic review and meta‐analysis [44] of 11 studies on CT radiomics predictions of MVI found that CT radiomics could serve as an efficient tool to predict MVI in HCC, with an AUC of 0.87. Notably, CT radiomics models based on 3D region segmentation and deep learning exhibited preferable accuracy compared to 2D segmentation and nondeep learning.

#### 3.2.2 MRI Radiomics

An MRI radiomics study found that the higher soft tissue contrast and additional imaging sequences of MRI provided more favourable predictive performance than CT [45]. The latest meta‐analysis [46] included 13 qualified studies of 3209 patients with HCC and achieved an average radiomics quality score of 14. In the final assessment, MRI radiomics showed a great capability to predict MVI, with pooled sensitivity, specificity and AUC of 82%, 79% and 0.88 respectively.

#### 3.2.3 PET‐CT Radiomics

PET radiomics has gained potential in predicting MVI in the past few years [47]. Sabat'e‐Llobera et al. [48] first proposed that the SUV peak (SUVmax) was the optimal parameter to predict MVI, which was estimated by a semiquantitative analysis of HCC lesions. The results were subsequently verified by Çelebi et al. [49], who reported that the SUVmax of PET radiomics could independently predict MVI, with a sensitivity of 75%, specificity of 97% and AUC of 0.896.

### 3.3 Predictive Models

Currently, researchers suggest that predicting MVI by combining clinical features, laboratory indicators and radiomics could further improve the accuracy compared to a single indicator [50, 51]. Thus, predictive models of MVI have developed rapidly, including linear regression‐based models, such as nomograms, risk‐scoring systems, online predictive calculators and the latest models based on AI algorithms [52, 53] (Figure 1). The accuracy and reliability of MVI predictions could be further enhanced by establishing a multi‐indicator model and introducing highly efficient algorithms [26].

#### 3.3.1 Nomogram

As a visual predictive model, a nomogram provides an individualised, evidence‐based and highly accurate risk estimation, which is convenient to utilise and facilitates MVI‐related decision‐making [50]. Lei et al. [14] first developed a predictive nomogram model of MVI in HCC within Milan criteria, which involved the tumour number, tumour diameter, tumour capsule, AFP level, PLT count, HBV DNA load and the typical dynamic pattern. The model showed great accuracy in predicting MVI, with a bootstrap‐corrected C‐index value of 0.81. A recent validation study performed by Sun et al. [54] further compared the predictive performance of four eastern and western nomogram models using an external cohort. Lei's work presented favourable predictive accuracy, as well as optimal predictive stability.

#### 3.3.2 Online Risk Calculator

Easy‐to‐use online calculators to predict MVI risk were developed in the latest research to further facilitate the clinical applicability of the predictive model [55]. Endo et al. [56] established an online calculator based on the AFP level, tumour number, tumour diameter and neutrophil and lymphocyte counts. The output of the online calculator was MVI probability, and it exhibited excellent accuracy and discrimination in predicting MVI in training and validation cohorts (C‐index = 0.72). Moreover, these easy‐to‐use online calculators could stratify HCC patients at high risk of MVI and recurrence and were helpful for recommending optimal treatment paradigms and postoperative surveillance.

#### 3.3.3 AI‐Based Predictive Model

AI has evolved in the last decade with the rapid expansion of data and is increasingly applied as an assistive tool for pathological diagnosis, medical imaging and clinical decision‐making [57]. According to the algorithm, it could be divided into nondeep learning (NDL) and deep learning (DL) models.

##### 3.3.3.1 NDL

NDL models commonly utilise LASSO and logistic regression to select features and implement a combination of radiomic and clinical features. NDL machine learning includes algorithms such as support vector machine, random forest and XGBoost [58]. Wang et al. [59] recently established a multiple machine‐learning fusion model based on gadoxetic acid disodium‐enhanced MRI and the gamma‐glutamyl transferase‐to‐platelet ratio to predict MVI in solitary HCC. They preliminarily built six machine‐learning models with great accuracy in predicting MVI in training, internal and external validation sets. Thereafter, an ensemble of all models (ENS) was established. This ENS model achieved the highest AUC in each set with excellent calibration and more net benefit.

##### 3.3.3.2 DL

Different from NDL, the DL has network model selection in addition to image slice selection due to the autonomous learning characteristics of its deep neural network. DL is based on deep network algorithms, such as convolutional neural network (CNN), back‐propagation neural network, multilayer perceptron and recurrent neural network [60]. The latest research reported that its ability to predict MVI status preoperatively was superior to the conventional linear models [61]. Chen et al. [62] developed an ‘interpretation‐based risk prediction’ method to predict the risks of MVI by integrating preoperative laboratory test results (i.e., blood parameters and AFP level) with DL techniques. The DL‐based models presented great accuracy in predicting MVI and showed outstanding performance with a C‐index value of 0.9052 in the validation cohort.

Radiomics and DL techniques have both shown desirable promising effects in predicting MVI; however, the differences and accuracy of the two methods are not well defined yet. Jiang et al. [63] compared the predictive accuracy of an XGBoost model (radiomics) and a 3D‐CNN model (DL) by estimating the same clinical database. The results showed the DL model was slightly more accurate than radiomics in predicting MVI (AUC: 0.906 vs. 0.887).

Currently, the major concerns associated with AI‐based models are their interpretability, generalisability and repeatability in clinical applications. No standardised protocols on how to report or interpret AI‐based results exist, nor is there a consensus on the feasibility of using AI for HCC patients [64, 65] Moreover, AI models require external validation to improve their accuracy and repeatability. Most published studies implemented training and validation of their models depending solely on internal datasets, which may overestimate their predictive capability. Opening clinical databases as sources for external validation would be essential for the future of the AI landscape [66].

## 4 Perioperative Management of MVI to Improve the Surgical Outcomes of HCC

**FIGURE 2:**
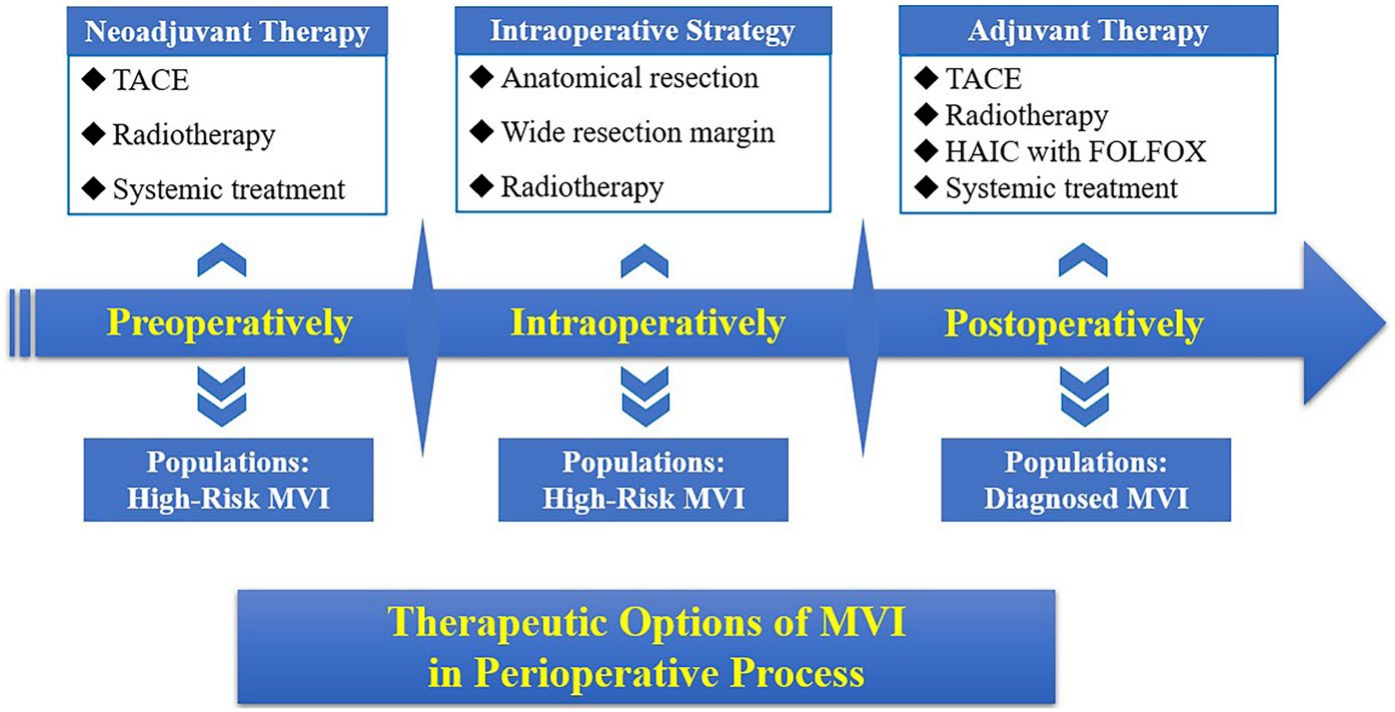
Therapeutic options of MVI in pre‐, intra‐ and postoperative processes.

Considering that the diagnosis of MVI is based on pathological specimens, the present management of MVI mainly focuses on adjuvant treatment, which may decrease the risk of recurrence after curative resection. With the rapid development of imaging techniques and predictive models for MVI, accurate preoperative MVI prediction provides opportunities for individualised perioperative strategies, including preoperative treatment, surgical planning and intraoperative decision‐making in patients at high risk of MVI. In the last decade, researchers have developed several preoperative and intraoperative strategies (Table 2), as well as adjuvant treatment options that could improve postoperative outcomes in patients with MVI or at high risk of MVI [71] (Table 3, Figure 2).

**TABLE 2:** Evidence from latest researches in the impact of neoadjuvant therapy and intraoperative decision‐makings on surgical outcomes of HCC with pathologically diagnosed MVI or high‐risk MVI.

Treatment	Research	Enrolled populations	MVI definition	Sample size of groups	Efficacy comparisons
**Neoadjuvant therapy**					
Neoadjuvant TACE	Yang et al. 2022 [16]	Multicentre retrospective study of resectable HCC and MVI	Pathologically diagnosed MVI	Neoadjuvant TACE (PR > 90%, 226) vs. No treatment (1233)	1‐, 3‐ and 5‐year OS: 97.2%, 81.4% and 63.2% vs. 88.4%, 69.2% and 41.6% (*p* < 0.001)
Neoadjuvant RT	Wei et al. 2023 [15]	Prospective clinical trial of single and small HBV‐related HCC and MVI	High‐risk populations of MVI	Neoadjuvant RT (30) vs. No treatment (30)	1‐, 2‐, 3‐ and 5‐year OS: 96.7%, 86.7%, 83.3% and 72.7% vs. 100.0%, 93.3%, 79.6% and 60.7% (*p* = 0.399) 1‐, 2‐, 3‐ and 5‐year DFS: 86.7%, 76.7%, 60.0% and 56.3% vs. 90.0%, 66.7%, 52.8% and 45.7% (*p* = 0.448)
**Intraoperative decision‐making**					
AR vs. NAR	Hidaka et al. 2020 [67]	Multicentre retrospective study of resectable HCC and MVI	Pathologically diagnosed MVI	AR (422) vs. NAR (124)	1‐, 2‐, 3‐ and 5‐year OS: 90.9%, 73.9% and 62.3% vs. 91.8%, 75.7% and 66.7% (*p* > 0.05) 1‐, 2‐, 3‐ and 5‐year RFS: 68.8%, 45.8% and 38.2% vs. 75.4%, 43.1% and 36.6% (*p* > 0.05)
Wide vs. narrow RM	Han et al. 2019 [68]	Multicentre retrospective study of resectable HCC and MVI	Pathologically diagnosed MVI	Wide RM (193) vs. Narrow RM (159)	Median OS: 93.5 vs. 69.2 months (*p* < 0.01) Median RFS: 53.1 vs. 37.5 months (*p* < 0.01)
Combined AR/NAR and RM status	Zhang et al. 2023 [69]	Multicentre retrospective study of resectable HBV‐HCC and MVI	Pathologically diagnosed MVI	AR with wide RM (715) vs. AR with narrow RM (387) vs. NAR with wide RM (568) vs. NAR with narrow RM group (295)	Median OS: 78.9 vs. 51.5 vs. 48.0 vs. 36.7 months (*P* < 0.001) Median RFS: 23.6 vs. 14.8 vs. 17.8 vs. 9.0 months (*p* < 0.001)
Intraoperative RT	Wang et al. 2022 [70]	Prospective study of centrally located HCCs treated with narrow‐margin hepatectomy	Pathologically diagnosed MVI	Intraoperative RT (59) vs. No treatment (65)	Intraoperative RT vs. No treatment 1‐, 2‐, 3‐year OS: 100.0%, 96.6% and 74.6% vs. 76.2%, 61.9% and 46.9% (*p* = 0.016) 1‐, 2‐, 3‐year RFS: 79.3%, 62.1% and 45.8% vs. 47.6%, 28.6% and 22.9% (*P* = 0.025)

Abbreviations: CI, confidence interval, DFS, disease‐free survival; HR, hazard ratio; MVI, microvascular invasion; NAR, nonanatomical resection; OS, overall survival; PR, pathological responses; RFS, recurrence‐free survival, RM, resection margin; RT, radiotherapy, TACE, transcatheter arterial chemoembolization.

**TABLE 3:** Evidence from latest researches in the impact of adjuvant therapy on surgical outcomes of HCC with pathologically diagnosed MVI.

Adjuvant treatment	Research	Enrolled populations	Sample size of groups	Efficacy comparisons
Adjuvant TACE	Xiang et al. 2024 [72]	Multicentre cohort study of intermediate and resectable HCC with MVI	Adjuvant TACE (109) vs. No treatment (137)	1‐, 3‐, 5‐year OS: 81.7%, 47.2% and 26.1% vs. 67.3%, 35.6% and 18.5% (HR, 1.438, 95% CI: 1.049–1.972, *P* = 0.023) 1‐, 3‐, 5‐year RFS: 42.0%, 27.2% and 17.8% vs. 31.8%, 18.2% and 8.7% (HR, 1.443, 95% CI: 1.089–1.914, *P* = 0.009)
Adjuvant RT	Shi et al. 2022 [18]	Randomised clinical trial of resectable HCC with MVI	Adjuvant RT (38) vs. No treatment (38)	1‐, 3‐, 5‐year OS: 100%, 89.5% and 75.0% vs. 100.0%, 68.4% and 53.7% (*p* = 0.053) 1‐, 3‐, 5‐year DFS: 92.1%, 65.8% and 56.1% vs. 76.3%, 36.8% and 26.3% (*p* = 0.005)
Adjuvant HAIC with FOLFOX	Li et al. 2022 [73]	Multicentre, phase 3 clinical trial of resectable HCC with MVI	Adjuvant HAIC (157) vs. No treatment (158)	Median DFS: 20.3 vs. 10.0 months (HR 0.59, 95% CI: 0.43–0.81, *p* = 0.001) 1‐, 2‐, 3‐year OS: 93.8%, 86.4% and 80.4% vs. 92.0%, 86.0% and 74.9% (HR 0.64, 95% CI: 0.36–1.14, *p* = 0.130)
Adjuvant immunotherapy (sintilimab)	Wang et al. 2024 [74]	Multicentre, phase 2 clinical trial of resectable HCC with MVI	Adjuvant sintilimab (99) vs. No treatment (99)	Median RFS: 27.7 vs. 15.5 months (HR 0.534, 95% CI: 0.360–0.792, *p* = 0.002)
Adjuvant combined therapy (atezolizumab plus bevacizumab)	Qin et al. 2023 [17]	Global, phase 3 clinical trial of resected or ablated HCC with high‐risk of recurrence	Adjuvant therapy (334) vs. Active surveillance (334)	Improved RFS in the intermediate results (HR 0.72, 95% CI: 0.53–0.98, *p* = 0.012)

Abbreviations: CI, confidence interval, DFS, disease‐free survival, FOLFOX, 5‐fluorouracil and oxaliplatin; HAIC, hepatic and arterial infusion chemotherapy; HR, hazard ratio; MVI, microvascular invasion; OS, overall survival; RFS, recurrence‐free survival, RT, radiotherapy; TACE, transcatheter arterial chemoembolisation.

### 4.1 Preoperative Treatments for Patients at High Risk of MVI

#### 4.1.1 Neoadjuvant Radiotherapy

Wei et al. [15] conducted a randomised clinical trial enrolling consecutive patients with resectable single and small (≤ 5 cm) HBV‐related HCC who were predicted to be at high risk of MVI and randomised them (1:1) to a neoadjuvant intensity‐modulated radiation therapy (RT) group and an upfront surgery group. Patients receiving neoadjuvant RT achieved an objective response rate of 25.0% with mild toxicity. The long‐term OS and disease‐free survival (DFS) were comparable between the neoadjuvant RT and upfront surgery groups, with 1‐, 3‐ and 5‐year OS rates of 96.7%, 83.3% and 72.7% versus 100.0%, 79.6% and 60.7% (*p* = 0.399), respectively, and 1‐, 3‐ and 5‐year DFS rates of 86.7%, 60.0% and 56.3% versus 90.0%, 52.8%, and 45.7% (*p* = 0.448) respectively. However, given the insufficient sample size (30 cases) of the study, further validation cohorts with more cases are still required.

#### 4.1.2 Neoadjuvant TACE

Although no studies have reported the impact of neoadjuvant TACE on the surgical prognosis of HCC patients defined as high‐risk MVI populations, several retrospective studies have explored the effect of neoadjuvant TACE on the incidence of MVI and the long‐term prognosis of patients undergoing curative resection of HCC [75]. Through multicentre database and propensity score matching analysis, Yang et al. [16] found that the pathological response (PR) ≥ 90% after TACE was an independent protective factor of MVI (odds ratio (OR) = 0.144, 95% confidence interval (CI): 0.082–0.245, *p* < 0.001), whereas PR < 60% was an independent risk factor of MVI (OR = 6.076, 95% CI: 3.004–11.728, *p* < 0.001). However, preoperative TACE was not associated with significantly improved long‐term survival compared to the control group.

### 4.2 Intraoperative Strategies for Patients at High Risk of MVI

#### 4.2.1 Resection Strategies

The influence of nonanatomical resection (NAR) or anatomical resection (AR) on the prognostic outcomes of HCC patients with MVI is controversial. A multicentre study from China [76] demonstrated that AR resulted in significantly better long‐term survival of patients with HCC and MVI than NAR. However, data from a large‐scale Japanese cohort [67] indicated that AR did not influence long‐term outcomes after hepatectomy in patients with HCC and MVI, but patients in the AR group had significantly decreased local recurrences around the resection site compared to those in the NAR group.

Regarding the optimal resection margin in patients with HCC and MVI, several clinical reports demonstrated that wide‐margin resection decreased recurrence risks in patients with MVI but not in patients without MVI [68]. Thus, a wide‐margin resection is expected to achieve superior recurrence‐free survival (RFS) in high‐risk MVI populations. It should be noted that wide‐margin resection needs to be considered more prudently and selected stringently, especially for patients with cirrhosis and multiple lesions, ensuring adequate remnant liver volume (RLV) before hepatectomy [77].

In terms of the best combination of NAR/AR and resection margin status, Zhang et al. [69] analysed a multicentre database of 1965 HBV‐related HCC patients with MVI, which first illuminated the impact of AR/NAR and resection margin status on the long‐term prognosis of HCC and MVI. Survival assessment identified that patients with HCC and MVI undergoing AR with a wide resection margin had significantly lower marginal recurrence rates, lower early recurrence rates and better long‐term outcomes than other groups.

The above clinical evidence indicates that AR with a wide resection margin is associated with optimal prognostic outcomes in patients at high risk of MVI based on the premise of adequate RLV. Further prospective clinical trials should be formulated to validate this hypothesis.

#### 4.2.2 Intraoperative RT

For centrally located and multinodular HCC in which achieving a wide‐margin resection is difficult, intraoperative RT is a potential therapeutic strategy for killing residual lesions and decreasing the recurrence rate in patients at high risk of MVI [78]. A prospective clinical study [70] explored the impact of intraoperative RT on the prognostic outcomes of HCC patients undergoing narrow‐margin hepatectomy. Intraoperative RT significantly improved RFS and OS in MVI‐positive HCC but not in MVI‐negative HCC. In detail, in the MVI‐positive subgroup, the 1‐, 2‐ and 3‐year cumulative RFS rates were 79.3%, 62.1% and 45.8% in the intraoperative RT group, and 47.6%, 28.6% and 22.9%, respectively, in the control group, with statistically significant differences (*p* = 0.025). These results suggest that prophylactic intraoperative RT may be an effective approach to decrease the risk of recurrence in selected patients at high risk of MVI and is worthy of further exploration.

### 4.3 Adjuvant Treatments for Patients Diagnosed With MVI

#### 4.3.1 Adjuvant TACE

TACE is currently one of the most commonly used adjuvant strategies after curative resection of HCC, with a relatively short treatment period and good safety [79]. It kills the underlying residual tumours released after surgery, as well as the existing microscopic tumours in the remnant liver, using localised chemotherapy and embolism. The current evidence suggests that adjuvant TACE can improve the outcomes of patients at high risk of recurrence, such as patients with multinodular HCC, pathologically diagnosed MVI and poorly differentiated HCC [80]. Recently, a large‐scale cohort study [72] demonstrated that among patients with intermediate and resectable HCC with MVI, liver resection plus adjuvant TACE was associated with significantly better RFS (hazard ratio (HR) = 1.443, 95% CI:1.089–1.914, *p* = 0.009) and OS (HR = 1.438, 95% CI:1.049–1.972, *p* = 0.023), compared with resection alone. In clinical practice, the optimal frequency of TACE (one time or multiple times) remains debatable. Some clinical studies [81] reported that multiple adjuvant TACE was superior to a single TACE among patients with early‐stage HCC and high recurrence risk by eradicating any residual occult intrahepatic satellite lesions not detected by imaging. However, other researchers [82] hold conservative opinions of intermediate‐stage HCC patients who may undergo the surgical removal of a large volume of liver tissue, which is associated with impaired liver and immune function. Thus, a single adjuvant TACE should be recommended for patients with intermediate‐stage HCC and MVI.

#### 4.3.2 Adjuvant Radiotherapy

Stereotactic body radiotherapy (SBRT) targeting the resection margin was suggested to act as a rescue procedure for MVI‐positive HCC patients after curative resection, as it could eradicate residual tumours and improve surgical outcomes [83]. Radiation applied precisely to the HCC bed area not only enhances the antitumour effect but also avoids exposure to the normal remnant liver. Moreover, the remodelling of the immune microenvironment induced by RT would also promote antitumour effects. Shi et al. [18] conducted a randomised clinical trial, enrolling a total of 76 participants with HCC and MVI undergoing curative resection. The 1‐, 3‐ and 5‐year DFS rates were 92.1%, 65.8% and 56.1% versus 76.3%, 36.8% and 26.3% in the SBRT group and surgery‐only group respectively (*p* = 0.005). In the safety profiles, no grade 3 or higher‐grade adverse events (AEs) occurred, and the overall incidence of RT‐related AEs was 31.6%. Another clinical trial [84] reported similar superior outcomes of adjuvant SBRT. In particular, in subgroup analysis, patients with a narrow resection margin acquired more survival benefits from adjuvant SBRT than those with a wide resection margin.

#### 4.3.3 Adjuvant HAIC

HAIC is another adjuvant local treatment used to kill residual HCC cells by elevating the localised concentrations of chemotherapeutic drugs. Several studies confirmed that HAIC in patients with advanced HCC had a higher response rate, with superior long‐term survival and tolerable toxicity than systemic chemotherapy. Japanese guidelines recommend it as a treatment option for advanced HCC [85]. In recent years, the prognostic significance of adjuvant HAIC in patients undergoing resection for HCC with MVI has stimulated research interest among clinicians. Li et al. [73] conducted a sequential multicentre, phase 3 clinical trial to substantiate the effect of adjuvant HAIC with 5‐fluorouracil and oxaliplatin (FOLFOX) in patients with HCC and MVI. HAIC in the FOLFOX group had a superior median DFS (20.3 months, 95% CI: 10.4–30.3 months) compared to the control group (10.0 months, 95% CI: 6.8–13.2 months). Another meta‐analysis involving 11 studies [86] also reported better long‐term surgical prognosis in the MVI subgroup of patients receiving adjuvant HAIC (*p* = 0.012), which could be an adjuvant strategy for HCC patients with MVI.

#### 4.3.4 Adjuvant Systemic Therapy

Tyrosine kinase inhibitors (TKIs) were shown to be one of the major adjuvant therapeutic options in previous investigations [87]. They target multiple kinases and proangiogenic receptors, thus blocking downstream signalling pathways and inhibiting the proliferation, angiogenesis and migration of tumours. Unfortunately, the STORM trial did not provide positive evidence for the efficacy of sorafenib as adjuvant therapy for HCC and MVI [88]. Immune checkpoint inhibitor (ICI) is another class of antitumour drugs to eradicate circulating cells by modulating regulatory signals between immune cells and generating long‐term responses [89]. Wang et al. [74] recently conducted a multicentre, randomised, controlled, phase 2 trial in China to assess the efficacy of adjuvant sintilimab, a programmed cell death protein 1 inhibitor, in HCC patients with MVI. Results at the prespecified endpoints showed that sintilimab significantly prolonged RFS compared to the surveillance group (median RFS: 27.7 vs. 15.5 months, *p* = 0.002). Subgroup analysis showed that the prognostic superiority of sintilimab existed even in patients with aggravated tumour burdens.

To date, no adjuvant systemic treatment has proven effective in global phase 3 studies. However, with the emergence of results from the IMbrave150 study [90], atezolizumab (antiprogrammed death ligand 1) plus bevacizumab (anti‐VEGF) has received research interest not only for patients with unresectable HCC but also for those with resectable HCC at high risk of recurrence. Qin et al. [17] conducted a global, phase 3 clinical trial (IMbrave050) to assess the outcomes of adjuvant atezolizumab plus bevacizumab versus surveillance in patients at high risk of recurrence (e.g., MVI and poor differentiation) after curative‐intent resection or ablation. The preliminary analysis showed significantly improved RFS in the treatment group compared to the surveillance group (median RFS: 22.1 vs. 21.4 months, *p* = 0.012) and a decreased recurrence risk of 28% in patients at high risk of recurrence.

### 4.4 AE Characteristics in Different Treatment Protocols

The incidence of AEs after adjuvant therapy is a significant factor in patient adherence to treatments, and it affects prognostic outcomes. As reported in recent clinical trials, the incidence of AEs in adjuvant loco‐therapy was lower than that of systemic therapy. The incidence of 0–1 grade AEs accounted for the majority of the AEs in adjuvant TACE, HAIC and SBRT, with rare 3–4 grade AEs. In contrast, the occurrence of 3–4 grade AEs in patients receiving adjuvant atezolizumab plus bevacizumab and adjuvant sintilimab reached 41.0% and 12.4%, with 9.0% and 8.1% of the participants withdrawing during the treatment respectively. The diversity in the incidence and severity of AEs may affect the selection of adjuvant therapeutic protocols for different populations.

## 5 Conclusion and Prospects

The present paper provides current advances in the diagnosis, classification, prediction and perioperative treatments to improve the prognostic outcomes of patients with MVI based on the latest research. The conclusions can be summarised as follows: **1**) The updated classification of MVI could better stratify patients at different risks of recurrence, facilitating postoperative surveillance and adjuvant treatment to prevent recurrence. **2**) With the incorporation of big data and AI, the performance of modern predictive models has become more accurate and individualised, which may guide neoadjuvant treatment options and intraoperative decision‐making. **3**) The perioperative treatment strategies for patients with high‐risk MVI or pathologically diagnosed MVI show favourable outcomes after curative resection for HCC. We emphasised regarding the classification, prediction and treatment of MVI into an integrity, instead of evaluating them separately. For instance, we summarised the current studies exploring the efficacy of preoperative treatment and intraoperative decision‐making for MVI, which utilised a well‐validated MVI predictive model, as criteria for patients at high risk of MVI [15]. Several classification systems incorporating multiple clinical variables are expected to guide optimal postoperative treatment [24, 25]. The integrity of these factors would facilitate the individualised and accurate management of MVI, improving the prognostic outcomes of patients with HCC and MVI.

However, challenges remain to be addressed in MVI‐related topics: **1**) In the diagnosis and classification of MVI, the sampling protocol and definition of MVI vary significantly around the world, making it hard to establish a standardised classification system. An MVI classification system could accurately discriminate patients with varied levels of postoperative recurrence, but its value in selecting optimal adjuvant therapy is unclear. Further classification combining pathological and clinical features may provide a rational scheme for the management of MVI. **2**) In the MVI prediction, it has undergone the process of predictive factors, predictive models and, now, the era of AI‐based models (Figure 1). However, several problems need to be solved concerning interpretability, standardisation, repeatability and generalisation of the predictive models. Using clinical databases as sources for training and validation would be essential for the future of the AI landscape. **3**) In the perioperative treatment of MVI, there are mostly retrospective studies with small sample sizes. Therefore, in the future, large‐scale clinical trials and real‐world studies of perioperative treatment for MVI should be conducted based on defining high‐risk MVI populations. Thus, integrating AI models into the entire process of MVI classification, prediction and treatment might be the next hotspot in MVI management.

## Author Contributions

**Zhenli Li:** data curation (equal), formal analysis (equal), writing – original draft (equal). **Lindi Xu:** investigation (equal), methodology (equal), writing – original draft (equal). **Shuaishuai Zhu:** software (equal), validation (equal). **Xingshun Qi:** writing – review and editing (equal). **Wei Zhang:** writing – review and editing (equal). **Yufu Tang:** conceptualization (equal), funding acquisition (equal), project administration (equal), writing – review and editing (equal).

## Ethics Statement

The authors have nothing to report.

## Consent

The authors have nothing to report.

## Conflicts of Interest

The authors declare no conflicts of interest.

## Data Availability

There are no original data in the review.
